# Estimation of Biomass and Canopy Height in Bermudagrass, Alfalfa, and Wheat Using Ultrasonic, Laser, and Spectral Sensors

**DOI:** 10.3390/s150202920

**Published:** 2015-01-28

**Authors:** Jeremy Joshua Pittman, Daryl Brian Arnall, Sindy M. Interrante, Corey A. Moffet, Twain J. Butler

**Affiliations:** 1 The Samuel Roberts Noble Foundation, Ardmore, OK 73401, USA; E-Mails: sminterrante@noble.org (S.M.I.); camoffet@noble.org (C.A.M.); tjbutler@noble.org (T.J.B.); 2 Oklahoma State University Department of Plant and Soil Sciences, Stillwater, OK 74078, USA; E-Mail: b.arnall@okstate.edu

**Keywords:** remote sensing, biomass estimation, mobile sensors, sensor system, data acquisition, high-throughput

## Abstract

Non-destructive biomass estimation of vegetation has been performed via remote sensing as well as physical measurements. An effective method for estimating biomass must have accuracy comparable to the accepted standard of destructive removal. Estimation or measurement of height is commonly employed to create a relationship between height and mass. This study examined several types of ground-based mobile sensing strategies for forage biomass estimation. Forage production experiments consisting of alfalfa (*Medicago sativa* L.), bermudagrass [*Cynodon dactylon* (L.) Pers.], and wheat (*Triticum aestivum* L.) were employed to examine sensor biomass estimation (laser, ultrasonic, and spectral) as compared to physical measurements (plate meter and meter stick) and the traditional harvest method (clipping). Predictive models were constructed via partial least squares regression and modeled estimates were compared to the physically measured biomass. Least significant difference separated mean estimates were examined to evaluate differences in the physical measurements and sensor estimates for canopy height and biomass. Differences between methods were minimal (average percent error of 11.2% for difference between predicted values *versus* machine and quadrat harvested biomass values (1.64 and 4.91 t·ha^−1^, respectively), except at the lowest measured biomass (average percent error of 89% for harvester and quad harvested biomass < 0.79 t·ha^−1^) and greatest measured biomass (average percent error of 18% for harvester and quad harvested biomass >6.4 t·ha^−1^). These data suggest that using mobile sensor-based biomass estimation models could be an effective alternative to the traditional clipping method for rapid, accurate in-field biomass estimation.

## 1. Introduction

An effective method for in-field estimation of biomass on a dry matter (DM) basis must produce accuracy comparable to the accepted measurement standard (*i.e.*, destructive removal). Non-destructive methods for estimating dry biomass using plant or canopy measurements have been developed [1,2]. In large part, vegetative mass is considered a function of canopy or plant height [3,4]. For these methods, canopy or plant height is recorded and an empirical relationship between height and DM is developed. Devices such as the rising plate meter, capacitance meter, and meter stick are examples of devices used for physical measurements of vegetation height and biomass estimation [1,5–7]. The limitations associated with these techniques are labor and time intensiveness. Additionally, variation due to vegetation growth characteristics and spatial variability can be difficult to accurately represent by physical sample collection which limits the ability to develop a robust estimation model.

Alternatively, remote sensing strategies (ultrasonic, laser, and sensor combinations) may overcome some of the limitations encountered with physical measurement strategies. A greater number of measurements can be taken in a considerably reduced amount of time and thus a larger area can be sampled. This increased magnitude in data collection provides opportunity for development of a statistically robust estimation model as a more comprehensive representation of the area of interest (AOI) can be collected. Ultrasonic proximity sensors employ intensity differential reflectance of sound waves to approximate distances. Ultrasonic sensors have been utilized for measuring height and estimating DM in pastures [2,8,9], canopy characterization in orchards [10,11], as well as in wheat [12], cotton [13], and maize [14]. Laser proximity sensors employ time-differential reflectance of light to approximate distances. Laser sensors have been effectively used for height measurements in wheat [15,16], maize [17], rape, rye, pasture [18], standing forests [19], and miscellaneous vegetation [20]. The combination of ultrasonic and active spectral reflectance have been used to estimate biomass in white clover, red clover, alfalfa, and perennial ryegrass, with R^2^ ranging from 0.90 for estimating alfalfa-perennial ryegrass mixtures to 0.99 for estimation of biomass for monoculture alfalfa [2]. Combining ultrasonic and Normalized Difference Vegetation Index (NDVI) measurements to estimate canopy height in wheat resulted in standard errors between 4.6 and 7.2 cm [12]. Similar results were found in maize using NDVI and ultrasonic sensors where an R^2^ = 0.62 was reported for forage mass [21].

Spectral strategies seek to base biomass estimates on reflectance or absorption intensities of wavelengths from vegetation and/or soil [2,22–24]. These spectral strategies can be effective at low Leaf Area Index (LAI) and biomass, but can become less accurate as the canopy closes when a point of reflectance saturation may occur [23,25]. Some direct contribution to reflectance saturation can be attributed to increase in vegetation height, but relationships between biomass and NDVI vary logarithmically, signifying an interaction of canopy closure and height [21,26]. Normalized difference vegetation index has been employed in biomass estimation for a number of crop species. Freeman [21] employed an active spectral sensor for calculating NDVI (Greenseeker^®^) and recorded a positive relationship with R^2^ = 0.52 for forage DM yield in maize. Erdle [22] reported R^2^ values of up to 0.91 and 0.84 for nitrogen content and biomass, respectively, when using active spectral sensors for NDVI measurement in wheat. Additionally, Gnyp [25] observed R^2^ of up to 0.69 for above-ground biomass in rice when regressed with NDVI alone, as well as a 21%–35% increase in explanation of above-ground biomass in rice when using a six-band spectral model as compared to NDVI alone.

There is limited published research on the combined use of ultrasonic, spectral, and laser sensors and their subsequent estimation models to measure forage height and DM. Therefore, the objective of this research was to evaluate the relationship between dry biomass measured via destructive removal and dry biomass estimated from a combination of sensor-measured canopy height and spectral reflectance. The evaluation was achieved by collecting both physically-measured and sensor-measured plant canopy heights as well as active and passive spectral reflectance readings via a mobile platform for vegetation at the canopy level. The intended deliverable from this research was a system containing a collection of sensors and software which would enable efficient and accurate acquisition of data by which estimation of dry biomass could be achieved.

## 2. Materials and Methods

### 2.1. Alfalfa-Bermudagrass Mixture Experiment: Site, Design, and Management

**Figure 1.**
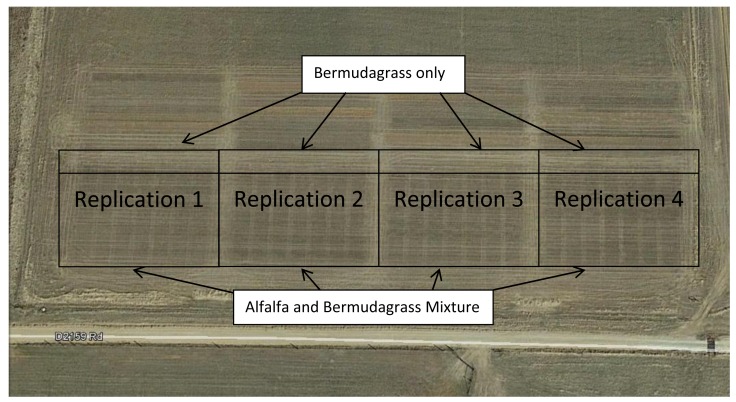
Aerial imagery of alfalfa and bermudagrass Trial. courtesy google earth.

A “600RR” alfalfa-“Midland 99” bermudagrass mixture trial was conducted at the Noble Foundation Red River Research and Demonstration Farm near Burneyville, OK, USA (33.88° N, 97.28° W; elevation 234 m. Figure 1). The soils are characterized as Slaughterville fine sandy loam (coarse-loamy, mixed, superactive, thermic Udic Haplustolls) with N-nitrate at less than 5 g·kg^−1^, soil test value of 64 g·P·kg^−1^, 52 g·K·kg^−1^ (amended with 0.1785 t·ha^−1^ 0-0-60), B of 0.017 g·kg^−1^ (amended with 0.00745 t·kg^−1^), and pH of 6.3.

A Hege 1000 cone planter no-till drill (Hege Equipment Inc., Colwich, KS, USA) was used for inter-seeding alfalfa into an established bermudagrass sward in fall 2012 and spring 2013. Data was collected the following spring and summer after establishment. Treatments were arranged in eight replications of a randomized complete block design (RCBD) with a split-split-plot arrangement. Main plots consisted of three alfalfa planting dates (September, November, and February), subplots consisted of three alfalfa seedbed preparations (mow/hay-off, mow/hay-off plus glyphosate, and tillage), and sub-subplots (1.5 m × 6 m) consisted of seven fungicide and insecticide alfalfa seed treatments. An adjacent experiment with eight replicates of 1.5 m × 6 m bermudagrass only plots treated with seven levels of N fertilizer ranging from 0 to 0.224 t·N·ha^−1^·yr^−1^ was established and harvested concurrently with the alfalfa-bermudagrass mixture experiment.

#### 2.1.1. Physical Measurements: Alfalfa-Bermuda Experiment

Data were collected from all plots four times in 2013 when alfalfa reached 10% bloom. Vegetation height measurements were taken using a meter stick and a 0.1-m^2^ aluminum rising plate meter (NZ Agriworks LTD t/a Jenquip, Feilding, New Zealand) [27,28]. Species composition was estimated both visually and as hand-separated dry weights from harvested quadrats for the alfalfa and bermudagrass mixtures only [29]. Visual composition was estimated and averaged across two observers [30]. Hand-separated alfalfa and bermudagrass subsamples were dried in a forced draft oven at 50 °C for five days to a constant weight prior to weighing. Plot biomass weights were recorded on a whole plot basis by clipping plots to a 5-cm stubble height with a Cibus forage harvester (Wintersteiger Inc., Salt Lake City, UT, USA) and are reported on a DM basis.

#### 2.1.2. Sensor Height, Spectral, and Spatial Measurements: Alfalfa-Bermuda Experiment

**Figure 2.**
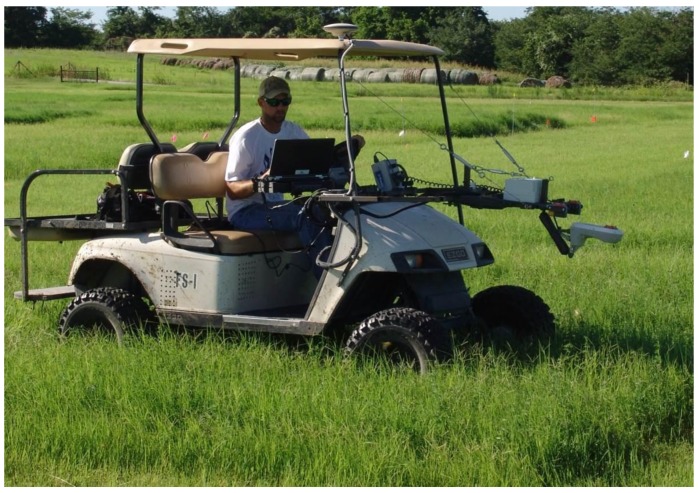
Electric golf cart used for transport of sensors across alfalfa-bermudagrass experiment.

A ground-based mobile platform was utilized for moving sensors across the trial areas using an electric golf cart (the golf cart was selected due to minimal suspension travel Figure 2) fitted with drop spindles and oversized tires spaced at 1 m, to minimize contact with the biomass contained within the plot area (1.5 m × 6 m). The cart was custom-fitted with a mast extending from the front upon which all sensors were attached. A single deep cycle 12 V DC marine battery was added to the cart and served as the power source for all sensors. Power and/or accessory power to all sensors was routed through a system power cycle switch by which all active data acquisition could be initiated or terminated simultaneously. Additionally, a GPS with Omni Star XP GNSS positioning (repeatability <10 cm, 95% CEP) was implemented to acquire position data for all sensor readings. The GPS was configured to output spatial data at a rate of 10 Hz such that multiple locations could be recorded within each plot. Height was measured using a single beam 660 nm time of flight laser distance sensor (“Laser”). The sensor was calibrated (calibration was performed prior to first use and verified by measurement at subsequent data collection events) to bare ground surface (0 cm) and 93 cm above ground surface as minimum and maximum heights, respectively. The laser readings were inversely related to height. The laser sensor used in this experiment differs from LIDAR laser systems which are typically aerial-based or ground-based static as opposed to mobile. Additionally, LIDAR laser systems typically scan a large area, utilize a large number of reflectance beams at numerous wavelengths, and they produce a “point cloud” [17,18]. The laser distance sensor used in these experiments emitted only one beam at one wavelength to produce a one dimensional pattern of measurements and did not have the multidimensional dynamics of a LIDAR point cloud. The laser readings characterize the height of the vegetation for a 2- to 4-mm diameter footprint which was inversely proportional to vegetation height. Two ultrasonic sensors, operating at different frequencies, were examined to observe appropriateness for use on plant material.

Height measurements were collected using a 240 MHz ultrasonic sensor and a 120 MHz ultrasonic sensor, which were also calibrated to ground height (0 cm) and 93 cm above the ground. Ultrasonic, sensor readings were directly proportional height. Readings from the ultrasonic sensors characterized canopy height in a 7.5- to 15-cm conical footprint, which were also inversely proportional to vegetation height. As the calibration for height sensors was 0–93 cm, all were operated within effective detection limits of near 0 m to 10 m specified by the manufacturer. All height data were acquired at the default sensor output rate (50 Hz–150 Hz) and configured to collect data centered on a 0.12 m^2^ area.

A Greenseeker^®^ (Trimble, Sunnyvale, CA, USA) was employed to collect NDVI with a maximum conical footprint of 0.1 m^2^ which varied inversely with forage height. Data were acquired at rate of 20 Hz from the Greenseeker^®^ radiometer for each plot. Additionally, a CROPSCAN (CROPSCAN, Inc., Rochester, MN, USA) with a conical footprint having diameter equal to on half of height to target was also used and acquired reflectance measurements at 450, 520, 530, 570, 590, 650, 690, 710, 780 and 900 nm (8.2–13 nm band width). Sampling using the CROPSCAN was limited to two readings per plot in an east/west travel pattern as sampling time was approximately two to three seconds per acquisition. There were fewer CROPSCAN than Greenseeker^®^ readings (two *vs.* 90 per plot, respectively) since each CROPSCAN acquisition event required a keystroke on the laptop computer compared to an automated acquisition from the Greenseeker^®^ sensor. Prior to data acquisition for the alfalfa and bermudagrass experiment the orientation of the two instruments were adjusted such that no reflectance from the Greenseeker^®^ radiometer influenced readings from the CROPSCAN.

#### 2.1.3. Transport and Temporal Logistics: Alfalfa-Bermuda Experiment

Each plot was driven across at 3.2–4.8 km·hr^−1^ resulting in approximately five seconds of data acquisition per plot. This amount of time resulted in approximately 25–30 condensed and balanced sample values per plot per parameter (*i.e.*, laser, GPS, Greenseeker^®^, *etc.*). After removal superfluous data associated with the power cycling, approximately four to five averaged readings per meter were assigned to each AOI. Rate of travel was dictated by the time necessary to acquire two samples with the CROPSCAN and the more extended data output time of this sensor as compared to others used. As previously stated, each sample required two to three seconds so five seconds were necessary to acquire both samples within the plot length. Length of data acquisition time per plot varied less than one second.

The sensor array measured approximately 25 cm wide and 45 cm from front to back with the Greenseeker^®^ radiometer located most forward and the ultrasonic proximal sensors located at the rear. The GPS was oriented approximately 75 cm to the rear of the ultrasonic sensors and approximately 45 cm right of center. This offset was accounted for in subsequent data parsing. The offset among sensors required that initiation and termination of sensor logging be timed to ensure that all vegetation measurement sensors were above the AOI while logging the data.

### 2.2. Wheat Experiment: Site, Design, and Management

**Figure 3.**
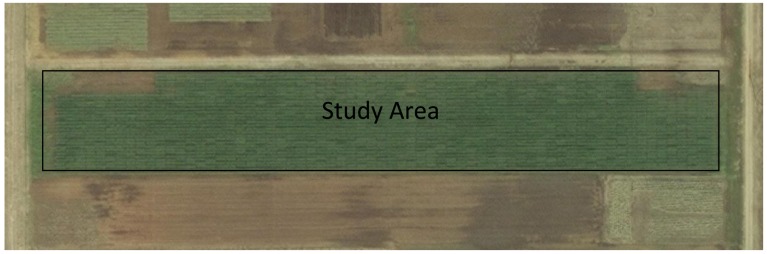
Aerial imagery of Dupy wheat experiment.

Two wheat trials were also employed for sensor data collection. The first wheat experiment was initiated at the Noble Foundation Dupy Farm near Gene Autry, OK, USA (34.29° N, 96.99° W; elevation 220 m. Figure 3). The soils are characterized as Dale silt loam with pH of 7.3 and N-nitrate, P, and K of 14, 31 and 132 g·kg^−1^, respectively. A Hege 500 cone planter grain drill (Hege Equipment Inc., Colwich, KS, USA) was used for planting wheat in autumn 2013, and data was collected in the spring 2014. Approximately 1200 (1.5 × 3 m) plots of 500 wheat varieties were planted as part of variety selection trials. These were arranged in completely randomized block design (CRBD) with two replications.

**Figure 4.**
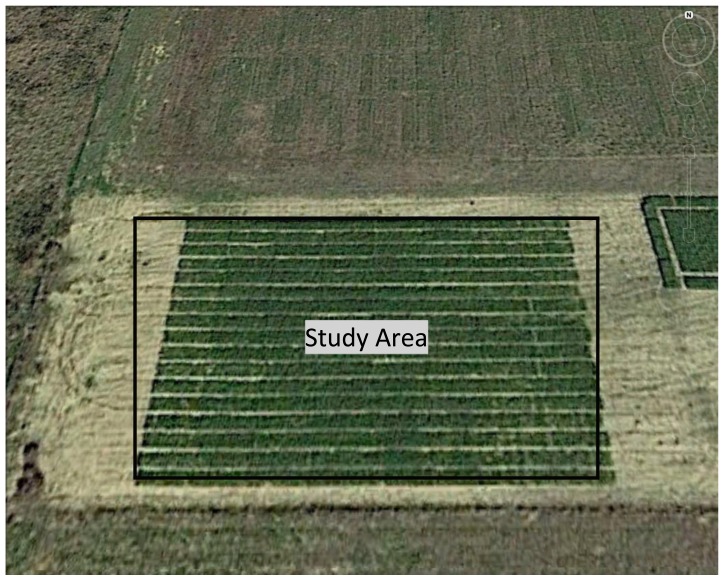
Aerial imagery of the unit 3 wheat experiment.

The second wheat experiment was initiated at the Noble Foundation Unit 3 Farm in Ardmore, OK, USA (34.17° N, 97.08° W; elevation 268 m. Figure 4). The soils are characterized as Konsil loamy fine sandy with pH of 6.8 and N-nitrate, P, and K of 28, 50 and 111 g·kg^−1^, respectively. This trial contained 136 (1.5 × 3 m) plots comprised of 50 wheat varieties. A Hege 500 cone planter grain drill was also used for planting wheat in 2013. Between the two wheat trials, there were seven data collection events occurring from February to April 2014.

#### 2.2.1. Physical Measurements: Wheat Experiment

Wheat biomass for both experiments was estimated by hand clipping one 0.16 m^2^ quadrat per plot to a 2.5-cm stubble height. Samples were dried in a forced draft oven at 50 °C for five days prior to weighing and are reported as kg·DM·ha^−1^.

#### 2.2.2. Sensor Height, Spectral, and Spatial Measurements: Wheat Experiment

**Figure 5.**
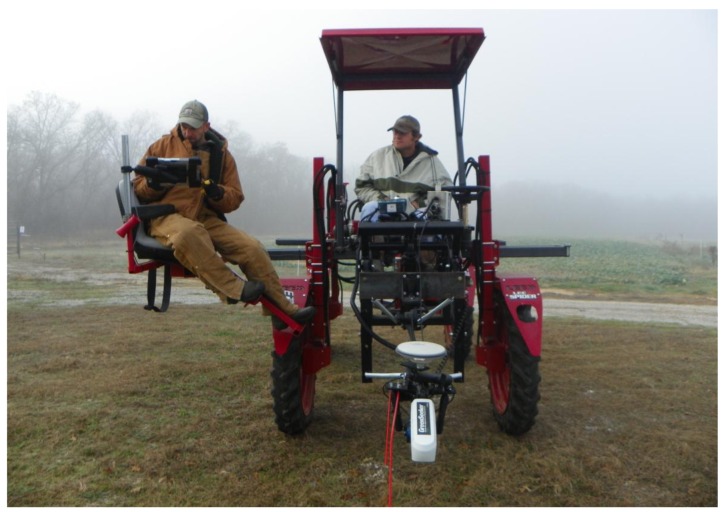
Spyder used for transport of sensors across wheat experiments.

Sensor data was collected from the wheat trials using a gasoline-powered Spider high-clearance tractor (LeeAgra, Inc., Lubbock, TX, USA) at a ground speed of approximately 1.6–3.2 km·h^−1^ (Figure 5). The factory-installed spray mast attached to the front of the tractor was converted to a manifold configuration to accommodate the sensor array. All sensors were initially powered using the onboard, factory installed, 12 V power supply. For convenience this was later modified to use an independent 12 V power source to power sensors. Upon restarting the tractor engine a momentary power deficit would occur and required re-initializing the sensor system. The same GPS with Omni Star XP GNSS positioning as described in the alfalfa and bermudagrass trial was implemented to acquire spatial data for all sensor readings. The GPS was configured to output data at a rate of 10 Hz such that multiple locations could be recorded within each plot.

Height was measured using two single 660 nm single beam time of flight laser distance sensors as well as a 120 MHz ultrasonic sensor. These sensors were the same make and model of those used in the alfalfa-bermudagrass experiment. All height sensors were calibrated at a bare ground surface and a 74 cm maximum in the same manner as those used in the alfalfa and bermudagrass experiment as well. All height data were acquired at a rate of 10 Hz and all sensors were configured to collect data centered on a 0.02-m^2^ area. A Greenseeker^®^ was employed to collect NDVI at rate of 10 Hz for each plot. The passive radiometer (CROPSCAN) was not used on the wheat experiments.

#### 2.2.3. Transport and Temporal Logistics: Wheat Experiment

The sensor array used in the wheat trials measured approximately 20 cm long by 10 cm wide. As was previously described in the alfalfa and bermudagrass experiment, it was necessary to ensure all sensors were above the plot prior to data acquisition initiation and termination. Alley identifiers were assigned during the time spent crossing the alley areas and until all sensors were above the subsequent AOI, whereas plot identifiers were assigned to the incoming data.

Each plot was driven across at 1.6–3.2 km·hr^−1^ resulting in approximately three seconds of data acquisition per plot and due to the fact a delay buffer of approximately one second was allowed at the beginning and end of each plot. This amount of time provided approximately 25–30 readings per plot per parameter (*i.e.*, Laser, Ultrasonic, Greenseeker^®^). Variability in the length of data acquisition time per plot occurred but was similar to the cart configuration in the alfalfa and bermudagrass experiment and only existed on the order of less than one second.

### 2.3. Data Acquisition Hardware: All Experiments

For all experiments, analog data from the height measurement sensors were acquired using a data acquisition module (DAQ). Laser sensors were configured via a current loop to operate at a range of 0 to 5 V DC and were connected directly to the DAQ for voltage output. The 120 MHz ultrasonic sensor was configured to operate at a range of 0 to 5 V DC, and voltage readings were directly output to the DAQ. The 240 MHz ultrasonic sensor was operated at a range of 0 to 10 V DC and voltage readings were directly output to the DAQ. From the DAQ, all analog data were transferred as digital output to a laptop computer for the alfalfa and bermudagrass trial and ruggedized tablet computer in the wheat trials via USB connection. Data from the Greenseeker^®^ radiometer was output directly to the tablet and laptop via serial connection as NDVI values, which were generated by the autonomous radiometer processor. CROPSCAN readings were acquired and stored in the autonomous memory contained within the radiometer hardware. It was not possible to insert plot markers into data from the CROPSCAN as it was necessary for the power source in this unit to remain autonomous and operational function could only be achieved through use of the factory provided software. However, it was possible to operate this software simultaneously on the same laptop, which was used for all other data acquisition.

### 2.4. Data Acquisition Software: All Experiments

For all experiments, all streams of data were captured real-time using AgriLogger and WinWedge Pro (WinWedge Pro^©^; TAL Technologies Inc., Philadelphia, PA, USA). These software applications were not used simultaneously for concurrent data collection. Both applications allowed for recording each stream of data with unique communication configurations. Unique configurations were necessary as retention of all data fields from all sensors was not desired and communication configurations for each stream of data or sensor were different.

Agrilogger was developed to utilize a sampling logic which resulted in only the user-specified rate of data acquisition to take place. All data was written to a single log file at the user-specified rate. In contrast, WinWedge Pro^©^ captured all data from incoming streams at rates dictated by the transmitting hardware. When using WinWedge Pro^©^, it was necessary to run multiple instances of the software simultaneously, one for each data stream (*i.e.*, DAQ, GPS, and Greenseeker^®^). Each instance of the software produced one log file for the data stream being acquired, which resulted in multiple output files for each data collection event.

The power cycling switch used for plot delineation when acquiring data with WinWedge Pro^©^ at no point interrupted data streams. The power was cycled on such that sensor readings were acquired and null readings were acquired when power was cycled off. These areas of null values signified non-plot areas. This strategy required combining the data from all streams post-processing. AgriLogger enabled the user to insert identifiers real-time as data were acquired. The identifiers used to delineate plot areas from non-plot areas were inserted with a mouse click or touch-screen button.

As previously stated, data acquired from the CROPSCAN were written to the autonomous storage capabilities contained within the unit. Utilization of factory included software application was necessary for post-processing reflectance data. This application produced one log file which could then be combined with data from all other sensors at post-processing based on sampling rate per plot and time stamp.

### 2.5. Data Post-Processing

For both WinWedge Pro^©^ and Agrilogger, time and date fields (based on the laptop clock) were inserted into the data streams for each record received at the application level. This allowed for quality control and the ability to combine data during post-processing when using WinWedge Pro^©^. Combining log files produced when using WinWedge Pro^©^ was achieved through implementation of a custom post-processing application (DataProcessing). The primary function of the DataProcessing application was averaging data to a desired rate (*i.e.*, 5 Hz, 10 Hz, *etc.*) so as to balance the number of sensor readings across the sensors and reduce the data to a more manageable volume. The averaging was achieved by utilization of the aforementioned time stamp which had been inserted for each record. After the data were combined, it was output to a text file which contained the combined sensor data at the specified averaging interval (*i.e.*, 5 Hz, 10 Hz, *etc.*). The output file from the DataProcessing software was then manually edited by attaching range and row identifiers to plot areas. These plot areas were delineated based on the aforementioned null values. The non-plot areas were manually removed from the data, leaving only the range and row identified plot areas. These plot areas could then be assigned a unique plot identifier.

Data acquired using AgriLogger was also manually edited to remove non-plot areas. Due to the user control and plot identification features provided in this software application, the magnitude of the data contained in the single output file were of a much smaller scale than that output by WinWedge Pro^©^. This was due to the sampling logic data acquisition strategy implemented in AgriLogger as opposed to the constant uncontrolled streaming of data with WinWedge Pro^©^. A user-specified sampling rate allowed for data acquisition at rates of up to 20 Hz and output of all parameters to a single log file. No post-processing application was necessary for reduction of the data through averaging.

Post-processing data transformation was performed on laser measurements as calibrations produced readings which were inversely proportional to the height of measured vegetation. Laser readings were subtracted from the calibrated maximum reading (signifying the greatest distance from the sensor). This transformed the data to a directly varying scale which agreed with the directly varying relationships between vegetation height and readings from ultrasonic sensors. Both ultrasonic and laser readings were converted to cm values based on the minimum and maximum calibration heights at post-processing.

### 2.6. Data Analysis: All Experiments

Sensor readings were examined for correlation to physically-measured height and destructively-measured DM [31–33]. The combination of output from multiple sensors as constituents of a predictive model for biomass was also examined. Comparisons were examined for the accuracy in estimation of height and DM for sensor models *versus* physical measurements performed. It must be noted that destructive harvest methods differed between the alfalfa-bermudagrass and the wheat experiments. Due to the 2.5-cm difference in harvest height, data analyses for biomass were performed based on harvest method and species for model construction. Additionally, the relationship between forage harvester and quadrat measurements of forage biomass was examined by regression analysis using SAS PROC REG [34].

#### 2.6.1. Correlation: Sensor Measurements to Physical Measurements

Physical and sensor measurement methods were examined for correlation to vegetative mass on a DM basis as well as sensor measurements to measured canopy height. These analyses were performed using SAS PROC CORR [34]. Data used for model construction were included for the examination of correlation between sensor measurements and physical measurements.

#### 2.6.2. Model Construction

Data were split into model construction and validation sets. This division was implemented to ensure the entire range of the data would be represented in both. Two hundred-twelve samples were used to generate estimation models for alfalfa only, with a validation set containing 89 samples. Seventy-eight samples were used to construct the bermudagrass only model with 32 validation samples. Wheat biomass models were constructed from 193 samples and 97 validation samples. Since no physically-measured canopy height data was collected for wheat, no canopy height estimation models were generated. Canopy height estimation models for the alfalfa and bermudagrass trial were constructed on a vegetation composition category basis, which consisted of individual species (alfalfa, bermudagrass), mixtures of the two (MIX), and across the entirety of the alfalfa and bermudagrass experiment data (ALL). The MIX group of data from the alfalfa and bermudagrass trial was comprised of sampled verified plots having botanical compositions ranging from 80% alfalfa:20% bermudagrass to 20% alfalfa:80% bermudagrass, (*n* = 1002 for modeling and *n* = 581 for validation).

Estimation models were constructed using partial least squares (PLS) regression analyses (SAS PROC PLS) with CVTEST for selection of simplest models [34–36]. Laser and ultrasonic sensor outputs as well as NDVI from the Greenseeker^®^ and seven spectral bands from the CROPSCAN were examined for inclusion in sensor based biomass estimation models. Laser and ultrasonic data were examined in the same manner for canopy height modeling.

Parameters included in biomass and canopy height estimation models were selected by evaluation of Variable Importance Plot (VIP) values output from SAS PROC PLS using the plots = (parmprofiles vip) option, as a filter measure [37] (Table 1). These scores represent the contribution of a variable as a predictor due to the amount of variance explained and can be viewed as a percentage in relation to one another when a number of predictor variables are simultaneously examined [37]. All components with VIP scores less than 1 were deleted from the final models for biomass and canopy height [38–40]. This strategy was adopted in order to achieve an acceptable balance in estimate accuracy and model/sensor system complexity by excluding less contributive variables and equipment from the system.

Upon deletion of less contributive variables, PLS models were again constructed using cross-validation (CVTEST option) with a 20-fold block training set (CV block = 20) and the SAS default of 1000 permutations [34]. Subsequently a randomly selected block of twenty observations provided cross validation analyses for model training 1000 times for construction of each model. The parameter estimates produced from these analyses were then employed in an equation for calculation of biomass and canopy height estimates (Table 2). Botanical composition based canopy height models included the laser and the 120 MHz ultrasonic (VIP scores > 1).

#### 2.6.3. Evaluation of Accuracy in Model Estimation for Biomass and Canopy Height

Regression analyses for canopy height and biomass estimations were performed to evaluate relationships between measured and estimated values using SAS PROC REG for samples from the validation data only [34]. Additionally, accuracy of estimation models was evaluated on a percent basis by calculating the by sample mean percent error (MPE) (Equation (1)):

(1) $$\text{MPE}=\frac{100\%}{n}\sum_{t=1}^{n}\frac{E_{t}-M_{t}}{M_{t}}$$

where *E_t_* is the biomass estimate for the *t*^th^ plot using the method of interest, *M_t_* is the measured biomass of the *t*th plot using the harvest method (*i.e.*, the accepted standard), and *n* is the number of plots measured.

An Error, Consistency, and Mean Agreement (ECMA) scoring system was calculated for ranking and comparison of model estimation accuracy. Calculation of this scoring system considered agreement of measured and corresponding estimated means, the by sample error estimation, and the repeatability of the error across samples in a category. The ranking calculation was compiled such that higher index scores represented more accurate estimation based on mean agreement, error, and the consistent nature of the error. The score included model error consistency (standard deviation of percent error, S_PE_), accuracy (mean of by sample percent error, MPE), and the agreement of the mean of measured compared to mean of estimated values (R^2^ of estimate to measured, as well as difference in mean of estimate “E” and measured “M”) (Equation (2)):

(2) $$\text{ECMA}=\frac{\left(\frac{r^{2}}{\left|Ē-\bar{M}\right|}\right)}{\text{MPE}\times s_{PE}}$$

Least significant difference (LSD) groupings (α = 0.05) were compared among plots grouped according to destructively-measured biomass and physically measured canopy height using the biomass and canopy height estimation models as a post-hoc analysis of accuracy (validation samples only were used in this comparison). This was done to illustrate the efficacy of using biomass or canopy height estimations calculated from sensor readings in place of destructive harvesting methods or physical height measurements for trial evaluations. Biomass comparisons groups were delineated in 1.10 t·ha^−1^ increments from 0 to 7.72 t·ha^−1^. Canopy height comparisons were based on 10 physically-measured canopy height classes at ten centimeter increments. These comparisons were performed using PROC MIXED [34] in combination with the PDMIX800 macro [41,42].

## 3. Results and Discussion

### 3.1. Correlation

Laser-estimated height measurements were the most correlated to physically-measured canopy height for all examinations (R = 0.88 bermudagrass to 0.78 MIX). Laser measurements were the most correlated to destructively-sampled biomass as well (R = 0.88 bermudagrass to 0.80 alfalfa). Additionally, NDVI measured using the Greenseeker^®^ was most correlated to biomass (R = 0.75 − 0.62) for all spectral data examined (Table 3).

Additionally, regression analysis of the harvester collected to quadrat collected biomass measurements yielded results showing quadrat harvests to produce AOI DM estimates on average 5% greater than measurements acquired using the harvester.

### 3.2. Modeling Analyses

#### 3.2.1. Variable Inclusion Selection

All height sensor parameters were associated with VIP values greater than the exclusionary threshold of 1 [37–40] for both biomass and canopy height (Table 1). The VIP values were greater for the 120 MHz ultrasonic sensor than for the 240 MHz ultrasonic sensor. The 120 MHz ultrasonic sensor was subsequently selected for inclusion in biomass and canopy height model construction. It was apparent while post processing data from the 240 MHz ultrasonic sensor that large areas were resultant output due to loss of echo which likely diminished contribution of this device in enhancing model accuracy. It may be possible to realize more contribution from a 240 MHz ultrasonic sensor which operates in a manner that does not produce such influential quantities of superfluous data. The spectral component NDVI was selected in the biomass model based on VIP values. Data from the CROPSCAN were not employed in the construction of models due to lower VIP scores than that of NDVI from the Greenseeker^®^. It is likely data from the CROPSCAN were less contributive to the estimation of biomass as it was implemented from a mobile platform and data were acquired while moving across plots as opposed to a stationary orientation. The CROPSCAN was designed to be used as a stationary passive spectral radiometer but it was desired to, in this research, evaluate the potential contribution of the CROPSCAN as an element of a mobile system. It is likely additional spectral bands or indices may be contributive to modeling biomass similar to observations reported by Gnyp [25]. This would be expected whereas the sensor producing this data is an active sensor designed for high speed acquisition as opposed to a passive sensor designed for stationary use as with the CROPSCAN used in these investigations.

#### 3.2.2. Biomass and Canopy Height Estimation Model Performance

Laser-only models explained more variation in dependent variable (VDV) for bermudagrass biomass (78%) and wheat (74%) than did ultrasonic only models for biomass and canopy height (75% and 72%, respectively). Combination models which included laser and ultrasonic improved dependent variable variation explanation in all cases except for wheat biomass (75%) and bermudagrass canopy height (77%). Inclusion of NDVI for biomass modeling improved dependent variable variation explanation by an additional 1%. Biomass models based on physically-measured canopy height were more effective than others in explanation of dependent variable variation for alfalfa only (68.5%). Plate meter-based models were less effective than others for canopy height and biomass estimation (0% to 6.1% and 18% to 23%, respectively) (Table 4). The greatest R^2^ observed (0.85) for any estimated to measured biomass relationship was the dual height sensor combination for bermudagrass. Comparison of biomass model estimation based on ECMA scores showed that models which included both height sensors as well as NDVI were more accurate in all cases except for bermudagrass (Table 5). The smallest R^2^ observed (0.13 to 0.25) in all cases was for the plate meter models. The meter stick measured height model was ranked second for alfalfa biomass estimation and was the only instance of a physical measurement model being ranked in the top two for biomass estimation. Regression of estimated canopy height to measured canopy height using model 9 for bermudagrass produced the greatest R^2^ for all cases (0.84). Canopy height estimates in alfalfa and the legume-grass mixture produced R^2^ values of 0.61 or less (Table 5).

### 3.3. Post Hoc Comparisons

#### 3.3.1. Sensor Estimation Models and Measured Biomass/Canopy Height Comparisons

Sensor models consistently overestimated small destructively measured biomass values and underestimated large values. Order of mean estimates for measured biomass and sensor-estimated biomass agreed except in the grass-legume mixture. In this case of order inconsistency, only the greatest biomass categories were inconsistent with measured values (>5.51 t·ha^−1^). The three sensor model (model 1) consistently produced lower biomass estimates than did the dual sensor model (model 3) for all species (Table 6).

Sensor models consistently underestimated canopy height for taller measured values but produced estimates within 4 cm of the lowest measured height from models 8 and 9. The only exception in minimum canopy height estimation occurred in MIX model 8 estimates where the estimate was 10 cm lower than measured (Table 7).

The LSD values for biomass and height estimation models were greater than those for the measured values. These LSD values indicated more variation within estimates for both canopy height and biomass resulting in overlapping of mean estimate groupings in some cases. Due to the fact the groupings were based on measured canopy height and measured DM, those measured groups would be expected to express the lowest variation. Additional error is also likely introduced into the modeled DM mean estimates due to the fact that the destructively measured DM does not capture in entirety the vegetation being sampled by the sensors. This error is illustrated in the stubble height remaining after destructive harvest which is embedded in and accounted for by the sensor readings and subsequently inseparable from the sensor models and sensor based estimations. This type of error could account for instances of over estimation at low biomass levels. Radial growth expansion as vegetation matured as well as under canopy fill-in may account for instances of biomass underestimation.

#### 3.3.2. Biomass Estimation Using Physical Canopy Height Measurements

Canopy height models varied by model and species, generally overestimating at lowest biomass categories and underestimating at highest biomass categories (Table 6). Model 6 overestimated whereas model 5 underestimated alfalfa biomass. Both measured height models overestimated bermudagrass biomass except for the greatest measured biomass. Biomass estimates for the mixture for both measured height based models overestimated at lesser values (<2 t·ha^−1^) and underestimated at greater values (>5 t·ha^−1^). Plate meter biomass estimation model ordered mean estimates the same as measured for only the alfalfa and consistently overestimated low and underestimated large measured values.

#### 3.3.3. Destructive Biomass Measurement and Model Estimate Variability

Due to the variation associated with destructively-measured biomass, it is unlikely that an estimation strategy based on this method of measure could achieve accuracy or precision in excess of the method upon which it is based. This destructively-measured variability is illustrated by the R^2^ of 0.87 from paired visually identical bermudagrass plots included in Table 5. A number of factors lead to error associated with machine harvest examples of which include operator performance, height and type of vegetation, and weigh mechanism performance due to environmental variables. The use of a sensor array to estimate biomass is subject to none of the factors in the same way the machine harvest strategy would be. Subsequently, basing the estimation of biomass by sensor populated modeling when constructed from machine harvest weight data will introduce all error associated the machine harvest into the model as well as any error associated with the sensor system. This will suppress the accuracy of the modeling due to the fact the number of error terms contributing to the calculation of biomass are from both the machine harvest as well as any associated with the sensors.

## 4. Conclusions

Using mobile sensor systems for biomass estimation can enable a greater rate of data acquisition than manual canopy height or destructive sampling provided an appropriate software option for data acquisition is employed. Results from this study illustrate quantification of only the canopy height with ultrasonic and laser sensors can provide for biomass estimation models equivalent to and/or more effective than those which include spectral components. This is an important distinction as the cost associated with assimilation of an active spectral radiometer into such a system can greatly increase costs. An increase of approximately 1% in dependent variable variation explanation was contributed to the system at a cost in excess of US $4,000. In contrast, height sensors and a DAQ would only incur a total cost of approximately US $1,500.

Additionally, sensor estimates provide equivalent and/or superior estimates when compared to physical canopy height measurement and plate meter biomass estimation methods. It is arguable that the same is the case for collection of sensor-based canopy height data, though a maximum height threshold of accuracy is likely according to the physical limits and configuration of sensors used. Due to the commonly accepted nature of physically measured biomass estimates for research applications, sensor-based estimation strategies which utilize species differentiation in appropriate cases and ultrasonic/laser proximal sensor combinations have, in this research, been illustrated to produce comparable and/or more accurate results. Consideration should also be given to the time savings associated with using a mobile sensor system. During the course of these studies it was noted man-hours needed for physical collection of these data (30 h·rep^−1^) were greater by a factor of 60 than the time needed to collect data with the sensor system (0.5 h). Furthermore, processing of data acquired using AgriLogger reduced man-hour requirements by a factor of 10.

In order for the greatest level of accuracy to be obtained, it is likely necessary to implement specific models for predominant or monoculture species though a general estimation model may produce acceptable estimates for mixed species. It may also be possible to stratify implementation of models based on height measurement. This would allow adjustment of coefficients to accommodate minimum and maximum values which can be estimated imprecisely if only one model curve is applied to the entire range of canopy heights and biomass levels encountered. Further examination of spectral data as a model component may be necessary for other parameters not examined in these experiments. Future examination of additional species is also necessary to develop models for estimating DM across different environments and production systems.

Qualification of relative vegetative performance based on canopy height and/or biomass would also be possible and could contribute to variety selection for plant breeding. Difficulties in system calibration and sensor data conversion to absolute measures could be avoided in a qualitative system. It can also be asserted that results reported for research could be based on sensor-estimated biomass without the expectation of appreciable differences than would be reported from destructively sampled methods or physical measurement based estimates. Estimating biomass without vegetation removal would be useful for plant breeders needing to quantify biomass along with seed yield. In addition, forage mass could be measured prior to and post-grazing to evaluate persistence and production under grazing that currently cannot be done. Ultimately, real-world production management decisions such as stocking rate adjustments or forage harvesting intervals could be made in a much more rapid manner.

## Figures and Tables

**Table 1.** Variable Importance Plot (VIP) scores for sensor parameters considered for model inclusion ^†^ From Greenseeker^®^, ^‡^ From CROPSCAN.

**Sensor Measurement**	**VIP**	

	**DM**	**Canopy Height**
**Laser**	1.37	1.08
**120 MHz sonic**	1.32	1.01
**240 MHz sonic**	1.11	0.95
**NDVI** ^†^	1.10	
**690 nm** ^‡^	0.90	
**650 nm** ^‡^	0.83	
**710 nm** ^‡^	0.74	
**590 nm** ^‡^	0.74	
**450 nm** ^‡^	0.72	
**520 nm** ^‡^	0.71	
**570 nm** ^‡^	0.70	
**530 nm** ^‡^	0.69	
**780 nm** ^‡^	0.68	
**900 nm** ^‡^	0.66	

**Table 2.** Dry biomass estimation model label key for sensor and physical measures.

**Model Number**	**Estimate**	**Species Specific**	**Number of Sensors**
**1**	Dry biomass	Y	3
**3**	Dry biomass	Y	2
**5**	Dry biomass	Y	Meter stick
**6**	Dry biomass	N	Meter stick
**7**	Dry biomass	N	Plate meter
**8**	Canopy height	Y	2
**9**	Canopy height	N	2

**Table 3.** Pearson coefficients for sensor collected parameters correlated to physical measures. ^†^ From Greenseeker^®^, ^‡^ From CROPSCAN.

**Sensor Measurement**	**Alfalfa**	**Bermudagrass**	**Mix**	**Wheat**

**Destructively measured DM**				
**Measured height**	0.83	0.83	0.82	
**Plate meter**	0.43	0.48	0.43	
**Laser**	0.80	0.88	0.86	0.86
**120 MHz sonic**	0.74	0.87	0.85	0.85
**240 MHz sonic**	0.75	0.81	0.66	
**NDVI** ^†^	0.70	0.75	0.72	0.62
**450 nm** ^‡^	−0.35	−0.53	−0.41	
**520 nm** ^‡^	−0.33	−0.54	−0.36	
**530 nm** ^‡^	−0.32	−0.51	−0.32	
**570 nm** ^‡^	−0.36	−0.50	−0.37	
**590 nm** ^‡^	−0.41	−0.56	−0.38	
**650 nm** ^‡^	−0.48	−0.65	−0.51	
**690 nm** ^‡^	−0.47	−0.63	−0.54	
**710 nm** ^‡^	−0.45	−0.52	−0.27	
**780 nm** ^‡^	0.13	0.02	0.32	
**900 nm** ^‡^	0.08	0.04	0.28	

**Canopy height**				

**Plate meter**	0.11	0.25	0.24	
**Laser**	0.83	0.88	0.78	
**120 MHz sonic**	0.76	0.82	0.73	
**240 MHz sonic**	0.73	0.71	0.68	

**Table 4.** Explanation of variation in dependent variable (VDV) from partial least squares regression modeling for dry biomass and canopy height by sensor estimation models and model equations for height (combination ultrasonic and laser model) and biomass (two sensor model: laser and ultrasonic combination, and three sensor model: laser, ultrasonic and NDVI combination). ^†^ MIX-Mixture of alfalfa and bermudagrass; ^‡^ ALL-All monoculture and mixed species from the alfalfa and bermudagrass experiment.

	**Canopy Height**	**Plate Meter**	**120 MHz Ultrasonic**	**Laser**

**DM**	VDV	VDV	VDV	VDV

**Alfalfa**	68.5%	18%	55%	64%
**Bermudagrass**	69%	23%	75%	78%
**Wheat**			72%	74%
**MIX** ^†^	67.8%	19%	73%	73%

	**3 sensor model**	**3 sensor equation**	**2 sensor model**	**2 sensor equation**

**Alfalfa**	65.7%	(46.22 × Las) + 47.83(Son × NDVI)	65.5%	(46.9 × Las) + (43.13 × Son)
**Bermudagrass**	80.5%	(65.3 × Las) + 58.3(Son × NDVI)	81%	(65.7 × Las) + (49 × Son)
**Wheat**	75%	(118 × Las) + 108(Son × NDVI)	74%	231 × Las
**MIX**	78.9%	70.5(Las × NDVI) + 63.7(Son × NDVI)	78.5%	(61.3 × Las) +(53.9 × Son)

		**Plate meter**	**120 MHz ultrasonic**	**Laser**

**Canopy height**		VDV	VDV	VDV

**Alfalfa**		1.2%	57%	69%
**Bermudagrass**		6.1%	67%	77%
**MIX**		5.6%	54%	61%
**ALL** ^‡^		6.1%	55%	64%

			**2 sensor model**	**2 sensor equation**

**Alfalfa**			70%	(0.46 × Las) + (0.42 × Son)
**Bermudagrass**			77%	1.02 × Las
**MIX**			64%	0.017(Las × Son)
**ALL**			65%	(0.74 × Las) + (0.2 × Son)

**Table 5.** Sensor model ranking based on Error, Consistency, and Mean Agreement Score (ECMA) ^†^ MPE (0.19), Standard Deviation of MPE (0.36) and R^2^ (0.87) for measured were calculated from paired bermudagrass plots. ^‡^ MIX-Mixture of alfalfa and bermudagrass.

**Forage Type**	**Measure/Estimation Method**	**Mean**	**Mean-Percent Error (MPE** ^†^**)**	**Standard Deviation MPE** ^†^	**R^2^-Measured to Estimated** ^†^	**ECMA Score**

**DM (t ha**^−^**^1^)**						
**Alfalfa**	Measured DM	2.36				
**Alfalfa**	1	2.16	0.31	0.27	0.69	0.150
**Alfalfa**	5	3.02	0.30	0.24	0.63	0.083
**Alfalfa**	3	2.36	0.40	0.35	0.68	0.019
**Alfalfa**	7	2.16	0.53	0.67	0.13	0.005
**Alfalfa**	6	3.02	0.68	0.53	0.63	0.002

**Bermudagrass**	Measured DM ^†^	3.33	0.19	0.36	0.87	
**Bermudagrass**	3	3.41	0.32	0.57	0.85	0.069
**Bermudagrass**	1	3.45	0.36	0.6	0.81	0.033
**Bermudagrass**	6	3.54	0.36	0.55	0.82	0.022
**Bermudagrass**	5	3.98	0.47	0.65	0.82	0.005
**Bermudagrass**	7	1.9	0.63	0.69	0.25	<0.000

**MIX** ^‡^	Measured DM	2.15				
**MIX**	1	1.83	0.30	0.26	0.79	0.035
**MIX**	3	2.37	0.40	0.52	0.78	0.019
**MIX**	6	2.76	0.68	0.79	0.55	0.002
**MIX**	7	1.66	0.48	0.58	0.17	0.001
**MIX**	5	3.1	0.85	0.91	0.55	0.001

**Wheat**	Measured DM	2.47				
**Wheat**	1	2.74	0.41	0.65	0.81	0.013
**Wheat**	3	3.18	0.63	0.94	0.80	0.002

**Canopy height (cm)**						

**Alfalfa**	Measured height	30.8				
**Alfalfa**	9	22.5	0.30	0.17	0.61	0.04
**Alfalfa**	8	21.0	0.33	0.18	0.59	0.03

**Bermudagrass**	Measured height	36.1				
**Bermudagrass**	8	29.0	0.21	0.12	0.79	4.05
**Bermudagrass**	9	26.0	0.28	0.13	0.84	2.29

**MIX**	Measured height	28.2				
**MIX**	9	18.5	0.35	0.19	0.57	0.88
**MIX**	8	7.2	0.77	0.17	0.59	0.21

**Table 6.** Mean estimates and LSD groupings for destructively measured DM and sensor modeled estimates of DM. ^†^ MIX-Mixture of alfalfa and bermudagrass.

**Forage Class**	**DM Class**	**Measured DM**	**Sensor Estimate DM Models (t**·**ha**^−^**^1^)**			

	**(t**·**ha**^−^**^1^)**	**(t**·**ha**^−^**^1^)**	**1**			**3**
**Alfalfa**	>1.1	0.85E	1.28D			1.5D
**Alfalfa**	1.1–2.2	1.65D	1.86C			2.09C
**Alfalfa**	2.2–3.3	2.72C	2.66B			2.89B
**Alfalfa**	3.3–4.41	3.6B	3.26A			3.45A
**Alfalfa**	4.41–5.51	4.88A	3.81A			4.01A
**LSD**		**0.22**	**0.44**			**0.44**

**Bermudagrass**	>1.1	0.67F	1.45E			1.58D
**Bermudagrass**	1.1–2.2	1.7E	1.97E			2.08D
**Bermudagrass**	2.2–3.3	2.96D	2.89D			2.93C
**Bermudagrass**	3.3–4.41	3.77C	3.8C			3.67C
**Bermudagrass**	4.41–5.51	4.99B	5.2B			5.02B
**Bermudagrass**	5.51–6.61	6.97A	6.19A			6.53A
**LSD**		**0.53**	**0.75**			**0.84**

**MIX** ^†^	>1.1	0.83G	0.98F			1.53F
**MIX**	1.1–2.2	1.61F	1.26E			1.82E
**MIX**	2.2–3.3	2.67E	2.32D			2.84D
**MIX**	3.3–4.41	3.79D	3.02C			3.45C
**MIX**	4.41–5.51	4.92C	4.45B			5.03B
**MIX**	5.51–6.61	6.15B	5.06A			5.56A
**MIX**	6.61–7.71	7.06A	4.56B			4.94B
**LSD**		**0.15**	**0.28**			**0.27**
**Wheat**	>1.1	0.78F	1.51D			1.93D
**Wheat**	1.1–2.2	1.57E	1.79D			2.27D
**Wheat**	2.2–3.3	2.76D	2.81C			3.26C
**Wheat**	3.3–4.41	3.85C	4.33B			4.75B
**Wheat**	4.41–5.51	4.89B	4.98A			5.28B
**Wheat**	5.51–6.61	6.25A	5.48A			5.85A
**LSD**		**0.27**	**0.53**			**0.54**

			**Measured height DM Estimate models (t**·**ha**^−^**^1^)**			
			**5**	**6**	**7**	

**Alfalfa**	>1.1	0.85E	1.18D	1.81D	1.7D	
**Alfalfa**	1.1–2.2	1.65D	1.72C	2.65C	2.08C	
**Alfalfa**	2.2–3.3	2.72C	2.39B	3.67B	2.31C	
**Alfalfa**	3.3–4.41	3.6B	3.05A	4.68A	2.63B	
**Alfalfa**	4.41–5.51	4.88A	3.51A	5.38A	3.07A	
**LSD**		**0.22**	0.46	0.71	0.46	

**Bermudagrass**	>1.1	0.67F	1.62E	1.44E	1.6B	
**Bermudagrass**	1.1–2.2	1.7E	2.78D	2.47D	1.66B	
**Bermudagrass**	2.2–3.3	2.96D	3.53C	3.14C	1.73B	
**Bermudagrass**	3.3–4.41	3.77C	4.85B	4.31B	2.05B	
**Bermudagrass**	4.41–5.51	4.99B	5.63B	5.01B	2.34A	
**Bermudagrass**	5.51–6.61	6.97A	6.44A	5.73A	2.27B	
**LSD**		**0.53**	0.97	0.86	0.62	

**MIX**	>1.1	0.83G	2.23F	1.98F	1.49C	
**MIX**	1.1–2.2	1.61F	2.88E	2.56E	1.49C	
**MIX**	2.2–3.3	2.67E	3.26D	2.91D	1.9B	
**MIX**	3.3–4.41	3.79D	3.56C	3.17C	1.89B	
**MIX**	4.41–5.51	4.92C	5.46B	4.86B	2.22A	
**MIX**	5.51–6.61	6.15B	5.95A	5.3A	2.1B	
**MIX**	6.61–7.71	7.06A	5.38B	4.79B	2.12B	
**LSD**		**0.15**	0.36	0.32	0.25	

**Table 7.** Mean estimates for measured canopy height (cm) and sensor model estimates of canopy height (cm). ^†^ No height data occurred in this range for bermudagrass. ^‡^ MIX-Mixture of alfalfa and bermudagrass.

**Forage Class**	**Height Class (cm)**	**Measured Canopy Height (cm)**	**Model 8 (cm)**	**Model 9 (cm)**
**Alfalfa**	>15	12J	16CD	16DE
**Alfalfa**	15–20	17I	14D	15E
**Alfalfa**	20–25	21H	16D	18DE
**Alfalfa**	25–30	27G	16CD	18DE
**Alfalfa**	30–35	33F	22BC	22CD
**Alfalfa**	35–40	37E	20BCD	23CD
**Alfalfa**	40–45	43D	27B	28C
**Alfalfa**	45–50	47C	26B	29BC
**Alfalfa**	50–55	53B	32A	35AB
**Alfalfa**	55–60	57A	35A	38A
**LSD**		**2**	**6**	**6**

**Bermudagrass**	>15	14I	13D	11D
**Bermudagrass**	15–20	16I	13D	11D
**Bermudagrass**	20–25	21H	17CD	15CD
**Bermudagrass**	25–30	29G	24BCD	20BCD
**Bermudagrass**	30–35	31F	25BC	22BC
**Bermudagrass**	35–40	36E	28B	25B
**Bermudagrass**	40–45	42D	30B	27B
**Bermudagrass**	45–50†	*	*	*
**Bermudagrass**	50–55	54C	43A	39A
**Bermudagrass**	55–60	57B	47A	43A
**Bermudagrass**	60+	66A	51A	48A
**LSD**		**3**	**12**	**10**

**MIX** ^‡^	>15	12J	2F	13E
**MIX**	15–20	17I	3F	14E
**MIX**	20–25	22H	3F	14E
**MIX**	25–30	27G	5E	16D
**MIX**	30–35	32F	7D	19C
**MIX**	35–40	36E	6DE	19C
**MIX**	40–45	42D	7DE	21C
**MIX**	45–50	48C	22C	34B
**MIX**	50–55	52B	27B	38A
**MIX**	55–60	57A	31A	40A
**LSD**		**1**	**2**	**3**
